# Recurrent tuberculosis in Finland 1995–2013: a clinical and epidemiological cohort study

**DOI:** 10.1186/s12879-017-2818-6

**Published:** 2017-11-16

**Authors:** Virve Korhonen, Hanna Soini, Tuula Vasankari, Jukka Ollgren, Pieter W. Smit, Petri Ruutu

**Affiliations:** 1 Department of Health Security, National Institute for Health and Welfare, Helsinki, Finland; 2 Department of Pulmonary Diseases, Tampere University Hospital, Tampere, Finland; 3 School of Medicine, University of Tampere, 33014 Tampere, Finland; 4 Finnish Lung Health Association (Filha), Helsinki, Finland; 5 Faculty of Medicine, University of Turku, Turku, Finland; 6 Department of infectious diseases, Public health laboratory, GGD Amsterdam, Amsterdam, The Netherlands

**Keywords:** Tuberculosis recurrence, Tuberculosis epidemiology, Tuberculosis treatment, Tuberculosis

## Abstract

**Background:**

We investigated the epidemiology and prevalence of potential risk factors of tuberculosis (TB) recurrence in a population-based registry cohort of 8084 TB cases between 1995 and 2013.

**Methods:**

An episode of recurrent TB was defined as a case re-registered in the National Infectious Disease Register at least 360 days from the date of the initial registration. A regression model was used to estimate risk factors for recurrence in the national cohort. To describe the presence of known risk factors for recurrence, patient records of the recurrent cases were reviewed for TB diagnosis confirmation, potential factors affecting the risk of recurrence, the treatment regimens given and the outcomes of the TB episodes preceding the recurrence.

**Results:**

TB registry data included 84 patients, for whom more than 1 TB episode had been registered. After a careful clinical review, 50 recurrent TB cases (0.6%) were identified. The overall incidence of recurrence was 113 cases per 100,000 person-years over a median follow up of 6.1 years. For the first 2 years, the incidence of recurrence was over 200/100000. In multivariate analysis of the national cohort, younger age remained an independent risk factor at all time points, and male gender and pulmonary TB at 18 years of follow-up. Among the 50 recurrent cases, 35 patients (70%) had received adequate treatment for the first episode; in 12 cases (24%) the treating physician and in two cases (4%) the patient had discontinued treatment prematurely. In one case (2%) the treatment outcome could not be assessed.

**Conclusions:**

In Finland, the rate of recurrent TB was low despite no systematic directly observed therapy. The first 2 years after a TB episode had the highest risk for recurrence. Among the recurrent cases, the observed premature discontinuation of treatment in the first episode in nearly one fourth of the recurrent cases calls for improved training of the physicians.

## Background

Tuberculosis (TB) remains a major global health problem with estimated 10.4 million new TB cases worldwide in 2015. In 2013, 0.3 million TB cases were reported as recurrent [1]. After successful treatment, recurrent TB is estimated to occur in 0–14% of all TB patients within 1–3 years [2]. Recurrence of TB following treatment of an initial disease episode can occur due to endogenous re-activation with the same strain of *Mycobacterium tuberculosis* (relapse) or exogenous infection with a new strain (re-infection). In low-incidence countries, recurrence rates have varied between 0.4% and in a prospective clinical trial up to 6% [3–5]. The proportion due to re-infection has been reported to vary between 4 and 27% [3, 4]. In high-incidence countries the majority of recurrent cases, up to 77%, are caused by re-infection [6].

Finland is a low-TB-incidence (<10/100000) country since 2001, and in 2015 TB incidence was 5/100000 [7]. In 2015, 1% of TB cases had HIV infection. However, emerging challenges for the TB control program include gradually increasing resistance of *M. tuberculosis*, with 3% of all isolates multi drug resistant (MDR) in 2015 [7], concomitantly with a rapid increase in the proportion of TB cases occurring in immigrants [8]. Finland did not implement a comprehensive DOT (directly observed therapy) strategy in patient management until 2013 [9].

Our previous study shows, that in Finland more than 80% of recurrent cases during 1995–2013 were relapses [10]. The aim of the present study was to investigate in a national, population-based TB cohort the occurrence of recurrent TB and a few potential factors affecting the risk of recurrence in Finland during the years 1995–2013. In recurrent cases, we describe treatment regimens administered and treatment outcome in the first episode, and other potential factors affecting the risk of recurrence, in order to strengthen the TB treatment program in the changing epidemiologic environment.

## Methods

### Surveillance system and study population

The study population consisted of all TB cases in Finland reported from January 1, 1995, to December 31, 2013, to the National Infectious Disease Register (NIDR), maintained at the National Institute for Health and Welfare (THL). Clinical microbiology laboratories mandatorily notify new *M. tuberculosis* isolations to NIDR and submit isolates to the Mycobacterial Reference Laboratory (NRL) at THL for drug susceptibility testing (ethambutol, isoniazid, pyrazinamide, rifampicin and streptomycin). Physicians mandatorily notify to NIDR laboratory-confirmed cases of TB: the laboratory report of a positive test result to the clinician automatically includes a reminder to notify the case. Since 2007, also clinically diagnosed TB cases, when a decision to give a full course of TB treatment is made, are notified. Information on HIV positivity is obtained by linking data within NIDR. Data on the country of origin and the date of death were retrieved from the national population registry. Data from the different sources are automatically linked as a case by a unique person identifying number.

### Case definitions and data collection for the subgroup of recurrent cases

**Fig. 1:**
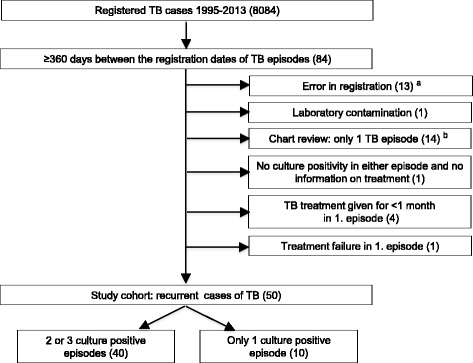
Steps in identifying recurrent cases of tuberculosis, Finland, 1995–2013. ^a^ Non-tuberculous mycobacterium registered as M. tuberculosis (*N* = 2), registration for false identity (*N* = 1) or incorrect registration date by the notifier (*N* = 10). ^b^ Only one long continuous TB episode (*N* = 5), or treating physician decided to give TB treatment without specific evidence of TB and in subsequent expert assessment by the study team there was another more likely cause for the disease (*N* = 9)

An episode of recurrent TB was defined as a case re-registered in NIDR at least 360 days from the date of the initial registration of a TB infection episode. For the cases who had a recurrent episode in the register, data on anatomical site of disease (pulmonary/extrapulmonary), radiological, histological and microbiological results, HIV test results, substance abuse, the drug regimen in the first episode and adverse effects were extracted from patient charts. In pulmonary TB cases, also sputum smear and culture results at months 0 and 2, and at the end of the treatment were obtained. Based on careful review of these data, a number of cases were excluded from further analysis as recurrent cases, as they did not meet the criteria for recurrent TB (Fig. 1). In culture negative episodes, the diagnoses were based on clinical criteria [11], including radiological findings in combination with either histological confirmation or positive nucleic acid amplification test results, except for one case in which the diagnosis was only clinical and radiological.

### Management of the TB episode preceding recurrence

The treatment regimen, free of charge for the patient, administered in the first TB episode was grouped into six categories, based on the national recommendations: until 2006 as described earlier [12, 13], and from 2006 in the National TB Control Program [9]. The treatment regimens and the outcomes of the first TB episodes of the recurrent cases, including culture negative and extrapulmonary cases, were assessed by two clinical TB experts, and classified [11] as cured, completed treatment, lost to follow-up and not evaluated (no fatal cases nor failures). ‘Lost to follow-up’ was further divided into subgroups: physician’s decision to stop prematurely (including cases that received ineffective treatment), and defaulted (interruption due to the patient). Group ‘not evaluated’ was further divided into subgroups: still on treatment at 12 months, and treatment outcome not assessed. In two cases the details of treatment in the first episode were not available, but in one of these cases the treatment outcome could be assessed.

### Statistical analysis

To calculate the incidence of recurrent TB among all reported cases of the national cohort, follow-up time in days was calculated for all cases in the national TB cohort from 360 days after they were notified until an event (re-notification), death or censored (December 31, 2013). The date of death was acquired by linkage to population register using person identifier. The regression model employed, uses pseudo-observations to model censored data [14] with Stata version 14.02 (StataCorp LLC, 4905 Lakeway Drive, Collage Station, TX 77845 USA) to estimate effects (their relative risks) of risk factors. The censoring was assumed to be independent, conditional on the covariates [15]. Gender and anatomical site of disease were allowed to have time dependent effects, and predictive margins were calculated to estimate cumulative risk differences between genders and anatomical sites of disease, respectively, adjusting for other factors in the model. Results were qualitatively checked using the extended time dependent Cox model. All the explanatory variables with univariate *p*-values <0.2 were included in the multivariate model, and included gender, anatomical site of disease and age. Cause specific cumulative risks for time points 1 year, 2 years and 18 years were calculated, as the chosen early time points had most recurrent events for explanatory variables, and it has been previously reported that the first 1–2 years have the highest hazard for recurrence [3, 16]. The maximum surveillance time point set at 18 years gives the final overall difference estimate for the cumulative risk of recurrence. The distribution of continuous variables between groups was compared using Wilcoxon rank-sum test.

### Ethics

The ethics approval for the study was given by the Ethics Committee of Tampere University Hospital, Tampere, Finland.

## Results

A total of 8084 TB cases were registered in Finland during the study period (Table 1): 43% were female, and 13.6% of foreign origin (increased from 4.8% in 1995 to 32.2% in 2013). The median age was 70 years (interquartile range [IQR], 56–79 years) for Finnish-born and 30 years (IQR 23–40 years, *p* < 0,001) for foreign-born cases.

**Table 1:** Distribution of demographic and potential risk factors for recurrent tuberculosis in a national TB cohort of 8084 cases, Finland 1995–2013

Variable	TB recurrence (*n* = 50)	No TB recurrence (*n* = 8034)	All (*n* = 8084)
Median age, years	51, 5	66	66
Gender female *n* (%)	11 (22%)	3457 (43%)	3468 (42.9%)
Foreign origin *n* (%)	9 (18%)	1066 (13.6%)^a^	1074 (13.6%)^b^
Pulmonary site of disease *n* (%)	44 (88%)^c^	5599 (69.2%)	5605 (69.3%)
Culture positive *n* (%)	48 (96%)^c^	6631 (82.5%)	6680 (82.6%)
Prior to year 2007 *n* (%)	45 (90%)^c^	5762 (71.7%)	5807 (71.8%)

^a^ Origin known for 7862 cases

^b^ Origin known for 7912 cases

^c^ Data of the first episode

### Characteristics and incidence of recurrent TB

After a careful review of the 84 cases with more than one episode registered, 50 TB cases (0.6% of all cases in the cohort) were classified as recurrent (Fig. 1). The mean overall incidence of recurrence was 112.9 (95% confidence interval [CI], 85.6–148.9); for the first year of follow-up the overall incidence was 236.4 (95%CI 140.0–399.2) and for the second year of follow-up 206.7 (95%CI 114.5–373.2) per 100,000 person-years. Out of the 50 recurrent cases, two had three disease episodes and 48 two disease episodes. Forty cases were culture positive in all episodes. The median age was 51.5 years (range 6–95 years) at the registration of the first episode; eleven cases (22%) were female. In the first episode 44 cases (88%) and in the second episode 39 cases (78%) were classified as pulmonary TB. Two cases were HIV positive; for 64% of cases HIV had not been tested. Nine recurrent cases (18%) were of foreign origin. A history of substance abuse, mostly alcohol, was registered in the patient records in at least 1 TB episode of 59% of males and none of females; 49% of males had substance abuse recorded in both episodes.

### Management of the TB episodes preceding recurrence

Among the 48 recurrent cases (96%) with complete patient records, 36 cases (75%) received standard treatment in the first episode (Table 2). Among these, the duration of treatment was short (<5.5 months) in six cases, and the intensive phase (<54 days) in three cases. Twelve cases (25%) received non-standard treatment due to drug resistance, adverse effects, or the treating physician’s decision. Of these, six cases received TB treatment regimens that were assessed as probably effective, one of them for a too short time period. Treatment regimens of six cases were assessed as probably ineffective. Six cases (12.5%) received directly observed therapy (DOT). Among the 49 cases with treatment outcome, it was successful in 27 cases (55.1%). In 12 cases (24.5%) the treating physician had stopped the treatment prematurely, and in two cases (4.1%) interruption was due to the patient. Eight cases (16.3%) were still on treatment at 12 months (all finally completed treatment).

**Table 2:** Distribution of treatment regimens in the first episodes of 50 TB cases with a recurrence

Treatment group	Total in group	Intensive phase short	Intensive phase adequate	Duration of treatment short	Duration of treatment adequate
Standard treatment A^a^	15	NA	15	1	14
Standard treatment B^b^	4	NA	4	2	2
Standard treatment with short intensive phase C^c^	3	3	NA	2	1
Standard treatment D^d^	14	0	14	1	13
Other probably effective combination of anti-TB drugs^e^	6	NA	NA	1	5
Other probably ineffective combination of anti-TB drugs^f^	6	NA	NA	NA	NA
Not evaluated	2	–	–	–	–

*NA* not applicable, *H* isoniazid, *R* Rifampicin, *Z* pyrazinamide, *E* ethambutol

^a^ HRZ in intensive phase, HR in continuation phase, adequate duration of treatment ≥  5.5 months

^b^ HRE in intensive phase, HR in continuation phase, adequate duration of treatment ≥ 8 months

^c^ Short intensive phase <54 days in standard treatment A or B

^d^ ≥  4 anti-TB drugs, including HRZ (adequate duration of treatment ≥ 5.5 months) or HRE (adequate duration of treatment ≥ 8 months)

^e^ Non-standard combinations guided by drug resistance or due to adverse effects, the adequacy of treatment duration assessed by the study group

^f^ Drug resistance ignored or inappropriate dosing

References [9, 12, 13]

There were no recurrent cases with an MDR isolate in either episode. Five cases were initially infected with isoniazid-resistant isolates, but in three of these cases, the treatment regimen was not modified accordingly. In two cases, additional resistance to streptomycin and in one case resistance to pyrazinamide developed during treatment. In one case, initially fully susceptible isolate developed resistance to pyrazinamide.

### Risk factors for recurrence in the national cohort

**Fig. 2:**
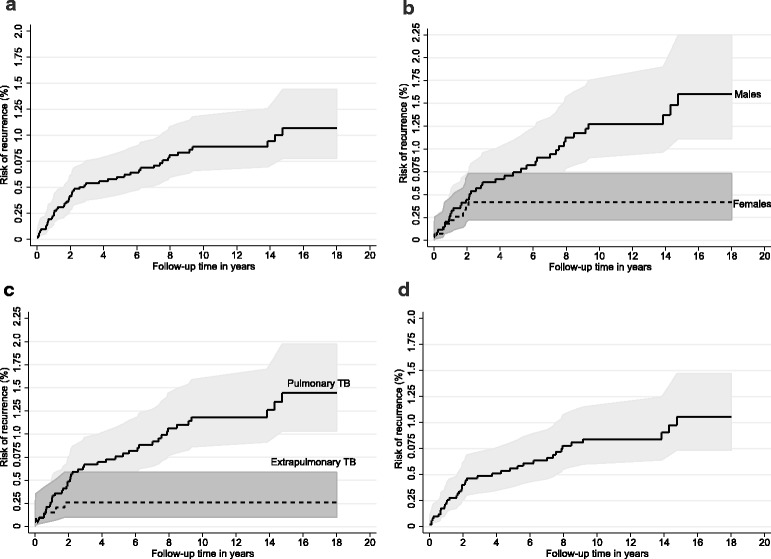
Cumulative risk of recurrence of TB by follow-up time (and its pointwise 95% confidence limits). **a** Overall cohort; (**b**) By gender; (**c**) By anatomical site of disease; (**d**) Only culture positive TB

The median follow-up time of cases in the cohort of 8084 TB cases was 6.1 years (IQR 2.7–11.1 years). The recurrence occurred within less than 2 years in 25 (50%), two to less than 4 years in 8 (16%), and later in 17 cases (34%) (Fig. 2a). No recurrences occurred in females and for extrapulmonary cases after the first 2 years (Fig. 2b and c). In univariate analysis of variables available for the national cohort, the cumulative risks of recurrence between males and females, and between pulmonary and extrapulmonary TB did not differ statistically significantly at 1 and 2 years of follow-up (Table 3). However, at 18 years of follow-up, the cumulative risk for males was nearly fourfold compared to females (Fig. 2b), and more than fivefold for pulmonary TB compared to extrapulmonary TB (Fig. 2c). The risk of recurrence decreased with every additional 10 years of age (Table 3). When only cases that were culture positive in all episodes were included, the recurrence rate was similar to that seen in the whole recurrent cases cohort (Fig. 2d). Whether the first episode occurred prior to versus after 2007 did not have a significant association with the risk of recurrence (Table 3).

**Table 3:** Univariate and multivariate analysis for risk factors of TB recurrence in a national cohort of TB cases in Finland, 1995–2013

Variables	Univariate RR	95% CI	*p*	Multivariate RR	95% CI	*p*
Males at 1 year ^a^	1,43	0,48–4,26	0,52	1,93	0,40–9,31	0,41
Males at 2 years ^a^	1,18	0,53–2,62	0,69	1,39	0,44–4,38	0,58
Males at 18 years ^a^	3,92	1,95–7,91	< 0,001	5,88	2,21–15,66	< 0,001
Pulmonary TB at 1 year ^b^	1,72	0,48–6,19	0,40	4,13	0,58–29,67	0,16
Pulmonary TB at 2 years ^b^	1,87	0,70–4,99	0,21	2,00	0,43–9,33	0,38
Pulmonary TB at 18 years ^b^	5,54	2,17–14,14	< 0,001	15,15	4,98–46,08	< 0,001
Age + 10 years	0,87	0,77–0,98	0,03	0,83	0,70–0,99	0,04
Finnish origin	0,64	0,31–1,33	0,23	–	–	–
1.episode before 2007	1,33	0,81–2,21	0,26	–	–	–

^a^ Reference females. Overall *p*-value for male gender in univariate analysis < 0,0001 and in multivariate analysis 0,0015

^b^ Reference extrapulmonary TB. Overall *p*-value for pulmonary TB < 0,0001

In the multivariate analysis (Table 3) younger age remained an independent risk factor for cumulative risk of recurrence at all time points, and male gender and pulmonary TB at 18 years of follow-up.

## Discussion

We investigated the epidemiology and the prevalence of risk factors associated with recurrence of TB in Finland in a comprehensive national, register-based cohort of 8084 TB cases from 1995 to 2013, and found in a follow-up of up to 18 years that 0.6% of cases during the study period were recurrent. The overall incidence of recurrence is 10–20 times higher, and for the first 2 years of follow-up 20–40 times higher than the incidence of TB in the general population in Finland during the study period [7]. Patient chart review of the recurrent cases revealed that in nearly one fourth of the recurrent cases, the physician had discontinued the treatment of the first episode prematurely.

In register-based studies on recurrent TB, validation of the data requires considerable effort to ensure data quality, as demonstrated in our study using a register with proven high sensitivity and specificity for TB [17], after which automated notification from the laboratories and mandatory reminders from the laboratory to the clinician on the need to notify were introduced. We observed that out of the 84 cases initially identified in the register data as recurrent, only 50 cases were truly recurrent. Major reasons for inaccuracies in our register data included incorrect notification dates, and notifying clinical TB cases without microbiological confirmation, which the chart review revealed to be incorrect. Without validation of the data for recurrent TB cases, the proportion of recurrent cases out of the total cohort in our study would have been almost double, stressing the need for rigid patient chart review when assessing TB recurrence.

Over 80% of recurrent cases in Finland are relapses of the previous infection [8], as elsewhere in low-incidence countries [3, 18, 19]. In register-based investigations of recurrent TB in low incidence-countries, careful review of the actual treatment in the first TB period of recurrent cases has rarely been reported [20, 21]. We observed that approximately two thirds, including those who received treatment lasting over 12 months, had received an adequate treatment. Just over one half among the recurrent cases had in the first episode a successful outcome according to WHO criteria [11]. Important for training policy was the finding that clearly more frequently than interruption due to the patient, the treating physician had discontinued treatment prematurely, as described in our previous report [12]. Inadequate treatments were caused by the presence of drug resistance without appropriate treatment regimen modification, absence of appropriate extension of duration of treatment when treatment was modified, adverse effects, or the physician’s decision to stop without the reason being documented in the patient record.

The overall incidence of recurrence in the national cohort, with a median follow-up period of 6 years, and up to 18 years, was 113/100000, 10–20 times higher than for the general population in Finland, in line with long-term follow-up in low-incidence countries (71–410/100000) [3, 16]. For the first year of follow-up, starting at 12 months from the registration of the first episode, the incidence of recurrence was 236/100000, of the same magnitude as in recent studies from Australia [3] and Denmark [18], but clearly lower than in a number of earlier studies from industrialised countries [22].

Our observation in the national cohort of male gender as a risk factor for recurrence is in line with some previous reports from low-incidence countries [23–25], but this finding has been inconsistent [20, 21]. Substance abuse data is not collected in the NIDR for the national cohort, but we found in nearly 60% of the recurrent male cases a history of substance abuse in patient charts, but none in females, which could contribute to the excess risk seen in males. An association between treatment adherence and alcoholism has been reported in recurrent TB in the USA [20]. We found in the national cohort the risk of recurrence higher for pulmonary than for extrapulmonary TB, in line with earlier studies [16, 23, 26]. More than 40% of our recurrent cases (data not shown) had both pulmonary and extrapulmonary infection in the first episode, which has been reported to be a risk factor for recurrences [25]. An unexpected finding in the national cohort was that the risk of recurrence was associated with younger age, whereas in two earlier studies, age > 65 years [20] or age between 25 and 64 years [23] have been reported as risk factors for recurrence. In earlier studies, either being an immigrant [16, 20, 25] or being borne in the country [21] were reported as risk factors, while origin was not a risk factor for recurrence in our national cohort study.

Limitations of our cohort study include the fact that we may fail to identify some recurrences, as recurrences before 360 days from the date of the initial episode (early recurrences) were not analysed from the register data. The standard cut-off time recommended by WHO, at which treatment outcome is recorded, is 12 months [11]. Therefore, we chose this timepoint as a cut-off for recurrence, in line with eg a large UK cohort [16]. The proportion of early recurrences in retrospective studies is small [3, 18]. In addition, in retrospective studies, it may be difficult to distinguish between treatment failures and early recurrences as sputum samples are not systematically collected during treatment, and in our study we also included culture negative and extrapulmonary cases. Almost one third of our recurrent cases do not meet the WHO treatment regimen description and outcome criteria for a recurrent case [11]. However, the careful validation process of our register data demonstrates that the same challenges are likely to be present in other register-based studies, unless careful validation has been performed. As a country with very low incidence for HIV [7], the absence of systematic testing of all TB cases for HIV is unlikely to introduce a bias in the risk analysis.

The observations on the shortcomings of treatment among the first episodes of the recurrent cases are important for guiding training and system development for the integrated TB control program. In 2013–2015, treatment outcome in Finland was successful (cure or completed treatment) in 75–78% of pulmonary TB cases [7], as in the European Region on average [27].

## Conclusions

In the absence of a comprehensive DOT strategy, the rate of TB recurrence was found to be low in Finland. An important finding was that in one fourth of the recurrent cases, the physician had discontinued the treatment prematurely, which implies that training of physicians needs to be improved and, as TB becomes rare, treatment should possibly be provided in fewer centers. The first 2 years after a TB episode is a very high-risk period for recurrence: this could be incorporated as an automated high-risk signal in the developing integrated electronic patient management systems for reducing the delays in implementing TB diagnostics and treatment.

## Abbreviations

CI: Confidence interval
DOT: Directly observed therapy
HIV: Human immunodeficiency virus
IQR: Interquartile range
MDR: Multi drug resistant
NIDR: National infectious disease register
NRL: Mycobacterial reference laboratory
TB: Tuberculosis
THL: National Institute for Health and Welfare

## References

[1] World Health Organisation (2015). Global tuberculosis report 2015.

[2] Cox HS, Morrow M, Deutschmann PW (2008). Long term efficacy of DOTS regimens for tuberculosis: systematic review. BMJ.

[3] Dobler CC, Crawford ABH, Jelfs PJ, Gilbert GL, Marks GB (2009). Recurrence of tuberculosis in a low-incidence setting. Eur Respir J.

[4] Jasmer RM, Bozeman L, Schwartzman K, Cave MD, Saukkonen JJ, Metchock B (2004). Recurrent tuberculosis in the United States and Canada: relapse or reinfection?. Am J Respir Crit Care Med.

[5] Kim L, Moonan PK, Yelk Woodruff RS, Kammerer JS, Haddad MB (2013). Epidemiology of recurrent tuberculosis in the United States, 1993-2010. Int J Tuberc Lung Dis.

[6] Verver S, Warren RM, Beyers N, Richardson M, van der Spuy GD, Borgdorff MW (2005). Rate of reinfection tuberculosis after successful treatment is higher than rate of new tuberculosis. Am J Respir Crit Care Med.

[7] Jaakola S, Lyytikäinen O, Rimhanen-Finne R, Salmenlinna S, Pirhonen J, Savolainen-Kopra C, Liitsola K, Jalava J, Toropainen M, Nohynek H, Virtanen M, Löflund J-E, Kuusi M, Salminen M. Infectious diseases in Finland 2015. THL, 2016. http://urn.fi/URN:ISBN:978-952-302-710-7. Accessed 7 Aug 2017.

[8] Raisanen PE, Soini H, Vasankari T, Smit PW, Nuorti JP, Ollgren J (2016). Tuberculosis in immigrants in Finland, 1995-2013. Epidemiol Infect.

[9] Ministry of social affairs and health. National tuberculosis control programme 2006. Ministry of Social Affairs and Health. Helsinki; 2006. http://urn.fi/URN:NBN:fi-fe201504225758. Accessed 7 Aug 2017.

[10] Korhonen V, Smit P, Haanperä M, Casali N, Ruutu P, Vasankari T (2016). Whole genome analysis of mycobacterium tuberculosis isolates from recurrent episodes of tuberculosis, Finland, 1995-2013. Clin Microbiol Infect.

[11] World Health Organisation. Definitions and reporting framework for tuberculosis: World Health Organization; 2013. http://apps.who.int/iris/bitstream/10665/79199/1/9789241505345_eng.pdf. Accessed 7 Aug 2017

[12] Vasankari T, Kokki M, Holmstrom P, Liippo K, Sarna S, Ruutu P (2007). Surveillance report: great diversity of tuberculosis treatment in Finland. Euro Surveill.

[13] Lääkintöhallitus. Tuberkuloosi ja sen lääkehoito. Finnish National Board of Health. Tuberculosis and its treatment. Kapseli 15. Lääkintöhallituksen julkaisu 1985. [In Finnish]. Helsinki: Finnish National Board of Health; 1985.

[14] Andersen PK, Perme MP (2010). Pseudo-observations in survival analysis. Stat Methods Med Res.

[15] Parner ET, Andersen PK (2010). Regression analysis of censored data using pseudo-observations. Stata J.

[16] Crofts JP, Andrews NJ, Barker RD, Delpech V, Abubakar I (2010). Risk factors for recurrent tuberculosis in England and Wales, 1998-2005. Thorax.

[17] Kokki M, Holmstrom P, Ruutu P (2005). High sensitivity for tuberculosis in a national integrated surveillance system in Finland. Euro Surveill.

[18] Bang D, Andersen AB, Thomsen VO, Lillebaek T (2010). Recurrent tuberculosis in Denmark: relapse vs. re-infection. Int J Tuberc Lung Dis.

[19] Interrante JD, Haddad MB, Kim L, Gandhi NR. Exogenous Reinfection as a cause of late recurrent tuberculosis in the United States. Ann Am Thorac Soc. 2015. doi:10.1513/AnnalsATS.201507-429OC. 10.1513/AnnalsATS.201507-429OC PMC4724895 26325356

[20] Selassie AW, Pozsik C, Wilson D, Ferguson PL (2005). Why pulmonary tuberculosis recurs: a population-based epidemiological study. Ann Epidemiol.

[21] Pascopella L, Deriemer K, Watt JP, Flood JM (2011). When tuberculosis comes back: who develops recurrent tuberculosis in California?. PLoS One.

[22] Panjabi R, Comstock GW, Golub JE (2007). Recurrent tuberculosis and its risk factors: adequately treated patients are still at high risk. Int J Tuberc Lung Dis.

[23] Kim L, Moonan PK, Heilig CM, Woodruff RSY, Kammerer JS, Haddad MB (2016). Factors associated with recurrent tuberculosis more than 12 months after treatment completion. Int J Tuberc Lung Dis.

[24] Pettit AC, Kaltenbach LA, Maruri F, Cummins J, Smith TR, Warkentin JV (2011). Chronic lung disease and HIV infection are risk factors for recurrent tuberculosis in a low-incidence setting. Int J Tuberc Lung Dis.

[25] Millet J-P, Orcau A, de Olalla PG, Casals M, Rius C, Cayla JA (2009). Tuberculosis recurrence and its associated risk factors among successfully treated patients. J Epidemiol Community Health.

[26] El Sahly HM, Wright JA, Soini H, Bui TT, Williams-Bouyer N, Escalante P (2004). Recurrent tuberculosis in Houston, Texas: a population-based study. Int J Tuberc Lung Dis.

[27] European Center for Disease Prevention and Control/WHO Regional Office for Europe (2016). Tuberculosis surveillance and monitoring in Europe 2016.

